# B Cell Responses upon Human Papillomavirus (HPV) Infection and Vaccination

**DOI:** 10.3390/vaccines10060837

**Published:** 2022-05-25

**Authors:** Priya R. Prabhu, Joseph J. Carter, Denise A. Galloway

**Affiliations:** Human Biology Division, Fred Hutchinson Cancer Center, Seattle, WA 98109, USA; pprabhu@fredhutch.org (P.R.P.); jcarter@fredhutch.org (J.J.C.)

**Keywords:** memory B cells (Bmem), long-lived plasma cells (LLPCs), anamnestic response, HPV vaccine

## Abstract

Infection with human papillomavirus (HPV) is the necessary cause of cervical cancer. Availability of vaccines against HPV makes it a highly preventable disease. HPV vaccines act through type-specific neutralizing antibodies produced by antigen-specific plasma cells known as long-lived plasma cells (LLPC). However, just as any other vaccine, success of HPV vaccine is attributed to the immunologic memory that it builds, which is largely attained through generation and maintenance of a class of B cells named memory B cells (Bmem). Both LLPCs and Bmems are important in inducing and maintaining immune memory and it is therefore necessary to understand their role after HPV vaccination to better predict outcomes. This review summarizes current knowledge of B-cell responses following HPV vaccination and natural infection, including molecular signatures associated with these responses.

## 1. Introduction

The fact that human papillomavirus (HPV) is a necessary cause of cervical cancer [1] and that there are effective vaccines against HPV, makes cervical cancer a highly preventable disease through vaccination. The three HPV vaccines available to date, Cervarix™ (bivalent-2vHPV) [2,3], Gardasil-4™ (quadrivalent-4vHPV) [4], and Gardasil-9™ (nonavalent-9vHPV) [5], are all widely studied and confer >90% (2v-and 4vHPV) and >95% (9vHPV) protection when administered prior to HPV exposure [6]. These vaccines provide the tools for elimination of morbidity and mortality due to cervical cancer and a proportion of genital (vagina, vulva, anus, and penis) and oropharyngeal cancers that are HPV-driven. The World Health Assembly passed a resolution calling for elimination of cervical cancer in August 2020 and WHO has launched a global strategy to accelerate the elimination of cervical cancer as a public health problem [7].

## 2. Vaccines—Mode of Response

Like most other prophylactic vaccines, HPV vaccines afford protection through production of antigen-specific antibodies some of which have the potential to neutralize the virus. Various clinical trials across the globe have accumulated evidence on antibody responses and subsequent protection following HPV vaccination with 2vHPV [8,9], 4vHPV [10,11,12], and 9vHPV [13,14] among healthy adolescents. Typically, the highest titers of antibodies are detected approximately a month after immunization and then wane for about 2 years, attaining a plateau which is maintained for years [15].

**Figure 1:**
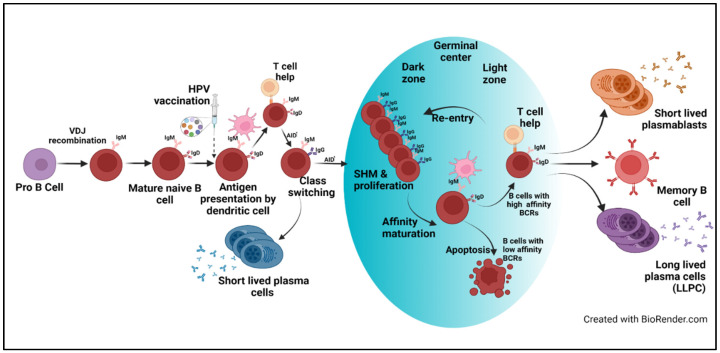
B cell activation, maturation, and proliferation upon exposure to antigens included in the HPV. Dendritic cells present HPV antigens included in the vaccine to naïve B cells. Binding to HPV antigens by B cell receptors results in B-cell activation and proliferation. Some B cells rapidly differentiate into plasma cells that secrete antibodies. B cells that receive additional signals from CD4^+^ T-follicular helper cells (TfH) express AID which is required for antibody class switching and somatic hypermutation (SHM) of antibody gene sequences. Germinal centers develop, containing activated B cells, activated TfH and dendric cells. It is in the light region of the germinal center that B cells compete for interaction with TfH, B cells with higher affinity receptors bind antigen and present peptides to TfH which in turn provide survival signals that promote further proliferation and continued SHM which takes place in the dark zone. B cells with low affinity receptors that do not receive survival signals undergo apoptosis. B cells can go through repeated rounds of SHM resulting in affinity maturation of the antibody genes, until cells exit as either short-lived plasmablasts, long-lived plasma cells, or memory B cells [Created with BioRender.com (Accessed from January to April 2022)].

Effective vaccines confer protection that either prevents infection (sterilizing immunity) or reduces disease through induction of immune memory mediated by B and T cells. Following primary vaccination, naïve B cells encounter an antigen that is recognized by the B cell surface receptor. Together with follicular dendritic cells and T cells, B cells form germinal centers in draining lymph nodes. In the germinal center, activation-induced cytidine deaminase (AID) is expressed, an enzyme that mediates somatic hypermutation of immunoglobulin genes and class switching [16]. Affinity maturation occurs as cells with higher affinity receptors replicate while cells with low-affinity receptors undergo apoptosis. This process results in short-lived, immunoglobulin-secreting plasmablasts, that are largely responsible for the peak of antibodies in the serum that occurs a week or two after infection/vaccination and long-lived plasma cells (LLPCs) that home to the bone marrow and secrete antibodies that can last for years to a lifetime (Figure 1) [17]. While most of the vaccines follow a similar process, some vaccines are much better than others at inducing long-lasting immunity.

Maintaining a steady supply of serum antibodies in circulation, the primary effector molecules for identifying and combating/neutralizing a pathogen, is achieved through their constant production by plasma cells. LLPCs are a class of terminally differentiated B cells, that secrete antigen/vaccine-specific antibodies and continue to do so for a long period, independent of additional antigen exposure [18]. Memory B cells (Bmem), on the other hand, are antigen-specific B cells, which are quiescent and long-lived, poised to recall and spontaneously respond to reinfection with the same antigen. Primary immune responses that occur after initial exposure to antigens in vaccines expand the pool of antigen-specific memory B cells, ‘priming’ the immune system and enabling immunized individuals to mount a rapid and robust response of higher magnitude upon re-exposure to the same antigen.

As a rule, infection and vaccination set off the production of large amounts of antibodies. However, not all antibodies are created equal. Neutralizing antibodies are those that can provide sterilizing immunity when experimentally transferred. However, some non-neutralizing antibodies have been shown to provide protection following certain viral infections [19,20]. There are also antibodies that provide little or no protection and some viruses appear to avoid elimination by tending to induce antibodies that bind but fail to protect. In a few rare instances, antibody responses result in a more severe disease [21]. We found that most antibodies that bind HPV16 also neutralize (unpublished data) which likely contributes to the high efficacy of the vaccine. Apart from the neutralizing activity of antibodies, Fc-dependent functions are required to clear the virus as well as virus-infected cells from the site of infection. Monoclonal antibodies targeted against the HPV L2 antigen have been shown to function via Fc-mediated phagocytosis of antibody bound HPV pseudovirus (PsV). A study conducted in a mouse model has shown that L2-specific monoclonal antibodies can cross the vaginal epithelium through micro-disruptions and that passive transfer of the F(ab’)2 region (Fc region cleaved off from whole IgG) showed lower protective capacity. Opsonization, through recognition of the Fc domain of antibodies bound to extracellular pathogens, tag them for phagocytosis by neutrophils and macrophages. However, since the F(ab’)2 without the Fc region failed to opsonize extracellular HPV PsVs, it could result in lower protective capacity. Similar results were seen in mice depleted of neutrophils and Gr1+ macrophages [22].

## 3. Techniques to Study B-Cell Responses

Antibody titers are considered the surrogates for B-cell responses. However, restricting to merely antibody testing would not provide a comprehensive measure of the B cell memory responses that are expected to occur upon re-exposure to antigen. Hence enumeration of B cells along with their functional consequence needs to be performed to successfully elucidate the recall response. One method to assess Bmem-cell response in a vaccine recipient is to re-expose the vaccinee with the same antigen or the etiological agent inducing the ‘anamnestic’ or memory response, characterized by a rapid spike in serum antibodies, proliferation of plasmablasts, and expansion of Bmems in circulation. One week after a vaccine booster dose, it has been estimated that upwards of 80% of plasmablasts are vaccine-specific [23] and are proportional to the preexisting levels of Bmems. Two weeks to a month after the booster dose, B cells in circulation can be isolated by antigen capture with phenotypic markers of memory B cells (CD19^+^, CD20^+^, CD27^+^, and IgD^−^) [24].

The ELISpot (enzyme-linked immune absorbent spot) assay is the most widely used test to quantify B cells (and T cells). In this assay, B cells, isolated from blood, are stimulated in vitro by exposure to the antigen of interest and 7–9 days later cells expressing antibodies or cytokines are enumerated [25]. Since the Bmems might migrate to lymphoid organs years after vaccination, the frequency of B cells measured in the peripheral blood may not be an accurate reflection of vaccine-specific Bmems. Moreover, HPV virus-like particle (VLP) specific Bmem ELISpot exhibited a high variability largely due to the low frequency of HPV-specific cells [26]. Any fluctuation from the extremely low number of spots (as low as 10 spots) leads to a large coefficient of variation. In addition, cellular manipulation required for the ELISpot assay adds further variability [27] and the B cells identified using ELISpot assay could not be used for any further downstream analysis. Nevertheless, as Bmems contribute to maintain levels of antibodies in circulation, Bmem ELISpot assays could be of great value in studies intended to understand the mechanisms involved in long-term maintenance of immune response after vaccination [23].

Our lab has previously developed an alternate method to detect HPV-specific Bmems by means of a combination of immunophenotyping, flow cytometry, and use of fluorescent pseudo viruses to bind receptors on surface of the Bmems [28]. The principal advantage of this method is that, in addition to quantifying the number of antigen-specific Bmems, antibody gene characteristics such as somatic hypermutation and class switching can be evaluated from single memory B cells. The amount of hypermutation after HPV vaccination was similar to that observed after other vaccines which, together with class switching, suggested B-cell development in the germinal center [28]. However, it was seen that a booster dose of HPV vaccine did not induce any higher levels of hypermutation in antibody genes, suggesting that either naïve B cells were being recruited or that further affinity maturation was not required [29]. Similar findings were observed for other vaccines such as SARS-CoV-2 vaccine [30,31]. Another advantage of this approach is that the antibody variable regions from individual Bmem can be cloned and expressed in vitro to evaluate their binding and neutralizing characteristics [28].

## 4. Evidence, Importance, and Comparison of B-Cell Responses across HPV Vaccines

Ideally, a vaccine achieves a long-lasting immune response ensuring long-term protection against the etiological agent. HPV vaccination is targeted at young adolescents to provide protection prior to sexual debut. This protection must be durable enough to last through early adulthood, during which time people are most sexually active [32]. This may perhaps be achieved through a persistent robust memory response upon HPV vaccination which could ensure protection for a longer duration, preferably without additional booster doses. Even though, in population-based interventions/studies, variables such as vaccine coverage, acceptance of vaccine, catch-up, etc., also contribute to vaccine efficacy, a long-lasting robust immune response and subsequent protection from disease among the vaccinees forms the very basis of effectiveness of any vaccine [33].

Studies from several research groups have demonstrated the memory responses upon HPV vaccination. All three HPV vaccines induced a spike in the number of circulating memory B cells. Similar to the serology results, HPV16-specific Bmem responses were greater than HPV18-specific Bmem responses one month post vaccination [34,35,36]. 2vHPV has been shown to induce antibody responses of higher magnitude than 4vHPV and Einstein et al. found a commensurate higher number of Bmem following 2vHPV vaccination compared to 4vHPV [34,35]. However, Nicoli et al. reported that the proportion of subjects (age > 16) with vaccine-specific memory B cells approximately 4–5 years post vaccination was higher among 4vHPV recipients (90%) than 2vHPV recipients (47%) [37]. Administration of a VLP-based HPV16 vaccine was shown to trigger the production of Bmems in 73.7% (1 month after the second dose) and 100% (1 month after the third dose) of the participants while none of placebo recipients had detectable Bmems [26]. A challenge dose with same vaccine, 5 years post-primary vaccination, engendered an anamnestic antibody response in recipients, demonstrating the existence of immune memory after 9vHPV [13] as well as 4vHPV vaccination [38]. These studies measured a rapid surge in antibody titer after a re-exposure, which is likely a surrogate for Bmem responses, providing functional evidence for effective generation of an immune memory post-vaccination. It is interesting to note that there were 2vHPV recipients with measurable antibodies in their serum without detectable Bmems, while in contrast, 4vHPV recipients without any detectable antibodies had Bmems in circulation. Yet, those vaccinated with 2vHPV had a higher number of HPV-18-specific Bmems than 4v HPV recipients. The anamnestic response elicited after a challenge dose 5 years later to primary vaccination with 3 doses of 9v HPV vaccine strongly suggests a robust immune memory which could be attributed to the presence of Bmems [13]. Subjects previously immunized with 3 doses of 2v vaccine, upon challenge with a fourth dose of the same vaccine 7 years later, demonstrated robust and rapid anamnestic responses [36]. The study reported that all subjects (100%) showed Bmem responses one month after a fourth dose of the 2v HPV vaccine, with ~55-fold and 15-fold increase in HPV 16 and 18 specific B cell numbers, respectively. Additionally, ~39- and 37-fold increases were observed in the HPV 31 and 45 specific Bmems respectively [36]. This could be attributed to the high amount of sequence similarity that HPV 16 shares with HPV 31 and HPV 18 with HPV 45. This establishes the possibility of three doses of 2vHPV to elicit a memory response not only against a targeted type, but also against closely related types. However, it has to be noted that circulating memory cells and other immune cells may not be an accurate indication of the size of the memory pool which has been shown to reside in peripheral lymphoid tissue and the immune effector cells at the site of infection and/or disease manifestation could be drastically different from what is observed in circulation [39,40].

## 5. Effect of Number of Doses, Recipient Age, and Type of Adjuvant in Vaccine-Induced B-Cell Response

Induction of optimal memory B-cell responses were observed in young children 9–13 years of age after 2 doses of 4v t vaccine when compared to 3 dose recipients, supporting a reduced (2-dose) regimen for young vaccinees [41]. However, this was not consistent with data from the older age recipients (16–26 years) who had a significantly reduced HPV 18-specific Bmem response after 3 doses when compared to the 3 dose recipients of younger age (9–13 years). This implies that age indeed influences immune memory and immunization between 9 and 13 years old would be advantageous to maximize HPV vaccine efficacy in terms of immune memory [41]. We have previously reported a lower Bmem response in terms of rate and magnitude following a third dose than after a booster dose (4th dose) given at 24 months after the vaccination series. This indicates that timing of the third dose is suboptimal and that dosing schedule for multidose vaccines should be determined based on the Bmem response that it elicits [29]. Timing between vaccine doses has a significant role in determining the long-term effect of the vaccine as it is known that B cells produced upon administration of the first dose of a vaccine require at least 4–6 months to mature and differentiate into high affinity memory B cells [42]. This suggests that the second dose should be administered after an optimal window period that allows affinity maturation of memory B cells and induces production and differentiation of B cells into antibody-secreting plasma cells and memory B cells that stay in circulation for a future recall response. The World Health Organization (WHO), through the position paper published in 2017, recommends a 2-dose schedule (0.5 mL at 0 and 5–13 months for 2VHPV and 9vHPV vaccines and 0.5 mL at 0 and 6 months for the 4vHPV vaccine) for girls and boys aged 9–14 years (2vHPV and 9vHPV vaccines) and 9–15 years (4vHPV vaccine). A third dose is recommended if age at the first dose is >15 (2VHPV and 9vHPV) or >14 (4vHPV vaccine) years. The 3 doses are to be administered at 0, 1, and 6 months for the 2vHPV vaccine, while for 4vHPV and 9vHPV vaccines are to be given at 0, 2, and 6 months. A third dose should be administered if the second dose is given earlier than 5 months (2vHPV and 9vHPV vaccine) or 6 months (4vHPV vaccine) [43].

Type and amount of adjuvants used in vaccines have an influence on the antibody responses. Whether or not this can be directly applied to the antigen-specific Bmem responses would be an interesting aspect to study. Giannini et al. demonstrated that the ASO4 (Mono phosphoryl Lipid A(MPL)/aluminum salt combination) formulation elicited a 2.2–5.2-fold higher Bmem response than aluminum-salt-only formulations [44], which is direct evidence for the influence of adjuvants on B-cell responses. MPL is a detoxified derivative of lipopolysaccharide (LPS) molecules from *Salmonella minnesota* that has immunomodulatory properties and can stimulate innate immune responses through prolonged activation of antigen presenting cells (APCs). This prolonged activation of APCs could influence the maintenance of plasma cells that contribute to a higher magnitude of circulating antibody response in 2vHPV vaccinees.

## 6. Molecular Signatures/Patterns/Properties of HPV-Specific B-Cell Responses

It has been postulated that the repetitive presentation of epitopes on HPV virus-like particles (VLPs), which are the basis of HPV vaccines, are responsible for inducing a strong and durable humoral immunity [45]. A sustainable and robust humoral response elicited by the vaccine is not limited against the high-risk types (HPV 16 and 18), but also against the low-risk types (HPV 6 and 11) targeted by the 4v HPV vaccine [37]. All HPV vaccine studies to date demonstrate a strong antibody response peaking a month after the final dose of vaccine, gradually declining to reach a plateau at around 2 years of vaccination. Since serum antibodies have a half-life of only a few days to less than a month [46], the long-lasting persistent antibody titers must be achieved through continuous replenishment by vaccine-specific LLPCs in an antigen-independent manner [47], rather than by Bmems. Had this long-sustained humoral response after HPV vaccination been a recall response by Bmems, a sudden surge of antibody titers is expected as and when a re-exposure to antigens occurs, which is not the case.

We have previously reported an inverse correlation between the Bmem responses and pre-existing Ab titers. Bmem responses one week after their 6-month dose (of a 0-, 2-, and 6-month dose schedule) were lower than when tested one week following an extra vaccine dose administered 2 years after primary vaccination. The high titers of vaccine induced antibodies after the first 2 doses of 4vHPV vaccine (at 0 and 2 months) still in circulation, may prevent the reactivation and proliferation of Bmems by neutralizing the vaccine antigen upon administration of the third dose, whereas after 2 years, when the titers of circulating antibodies decline, the additional dose promotes reactivation and proliferation of Bmems at a higher magnitude [29]. Similarly, in yellow fever vaccination with YF-17D vaccine, pre-existing neutralizing antibodies after a single priming dose were seen to be associated with the failure of YF-17D virus replication after a booster dose administered 10 years later [48]. Likewise, high levels of preexisting antibodies in circulation, to a given influenza virus strain result in production of lower levels of antibody-secreting cells and memory B cells recognizing the same strain upon re-vaccination. It is obvious to expect stronger serological response against those influenza strain antigens that are repeatedly administered across years. On the contrary, most robust B-cell response was observed against an antigenically divergent strain included in the vaccine compared to the repeated strain. This suggests that both preexisting memory B cell repertoire and levels of circulating antibodies influence the quality of the B-cell response to new prime-boost vaccine strategies [49].

Upon immunization with a monovalent HPV16 VLP vaccine, total HPV16-specific antibody titers and neutralizing antibody titers significantly correlated to frequency of Bmem responses, while no such correlation exists with the avidity index of the antibodies [26]. However, a study from Italy demonstrated that 2v- and 4vHPV vaccine-derived circulating antibodies and Bmems are independently maintained. The study reported that although vaccine-specific IgG titers and Bmems correlated immediately after vaccination, this correlation declines through the course of time which suggests that even though the vaccine induces both LLPCs and Bmems in early phases of immune response, they follow a different fate eventually [37]. A total agreement between the circulating antibodies and Bmems is not highly expected, as it is the LLPCs that contribute to continuous systemic supply of circulating antibodies and not the Bmems. Though it is still uncertain how the number of plasma cells are maintained for such a long time, there are two strong possibilities that could explain the mechanism of sustenance: (1) inherent long lifespan of LLPCs and (2) continuous replenishment of LLPCs by proliferation and differentiation of Bmems. The lack of correlation between circulating antibodies and Bmems during later stages of immune response suggest the possibility for long lifespan of LLPCs facilitated through various survival signals from the LLPC niche [50]. Under the latter hypothesis, a correlation between LLPCs (indirectly the measure of antibodies) and Bmems would be expected, which happens only during early stages of immune response. During early stages of immune response while somatic hypermutation and proliferation is still ongoing, it is expected that B cells would continuously replenish the plasma cells to maintain the protective antibody titers in circulation while in the later stages, those plasma cells which are terminally differentiated and genetically stable are maintained without additional antigenic stimulation and form the LLPCs. It is most likely that upon HPV vaccination, T helper cells through the production of cytokines support maturation of naïve B cell into effector cells such as LLPCs and Bmems, which helps in their sustenance for a long period [42,51]. Though evidence from animal studies suggests that T-cell-independent activation of naïve B cell populations is possible through innate stimulation [52], whether this happens in humans is unknown. The frequency of Bmems in circulation might not accurately reflect the overall frequency of HPV-specific Bmems as some may have migrated to secondary lymphoid tissue [39]. This could be another possible explanation for the lack of correlation between Bmem and antibody titers. Cloning and sequencing antibody genes provide insight into the evolutionary pathway followed from germline sequences to mature antibody [53,54,55,56,57,58,59,60,61]. This information has been used for other viruses to develop antigens designed to target B memory cells expressing specific heavy- or light-chain variable region genes [62]. Fortunately, the surface of HPV virus-like-particles is highly immunogenic so that approach is not necessary. HPV VLPs are composed of L1 proteins, which are highly conserved across HPV types. The variable regions in L1 that make them type-specific are interspersed among conserved segments and in the 3-dimensional structure they are displayed on the outer surface of capsomers embedded in five loops [63]. By analyzing the loops required for neutralization by human monoclonal antibodies, we found that all five L1 loops, that form the capsomer surface, were recognized by some mAbs. Most antibodies recognized conformational epitopes involving multiple surface loops (unpublished data). Vaccine-induced antibodies broadly recognize multiple epitopes (unpublished data) which could contribute to long-lasting immunity bestowed by HPV vaccination Recognition of multiple co-dominant epitopes by HPV antibodies is highly beneficial, as it would require a substantial number of mutations to escape antibody binding, which is highly improbable. This has recently been observed in the case of measles vaccine as well [64]. Neutralizing monoclonal antibodies from vaccinated subjects were encoded by many different heavy- and light-chain variable region genes; however, there were several antibodies that shared similar genetic sequences. These were found in the four subjects tested, but larger sequencing studies need to be conducted to know if these types of antibodies are induced by everyone.

## 7. B-Cell Response to Natural Infection with HPV

When considering the immune response to HPV infections, it is important to recognize that the virus lifecycle occurs entirely in the epithelium. By exploiting this anatomic niche, HPVs avoid expressing highly immunologic capsid proteins where they are readily accessible to immune cells, only expressing them in the upper layers of the skin. Thus, it is unsurprising that serum antibody responses following natural infections can be low or undetectable. Tissue resident immune cells are undoubtedly important for controlling and clearing infections but are not the subject of this article. Another consequential aspect of the HPV lifecycle is the slow rate of infection, which is a multistep process involving binding of L1 to heparin sulfate proteoglycans on the basement membrane, conformational changes to L1, and furin cleavage of L2 before cell entry. This process results in a delay of 12–24 h between virus exposure and viral transcription, during which time the virus can be neutralized by vaccine-induced antibodies. It has been proposed that the high efficacy of HPV vaccines can be explained by the viruses’ vulnerability during infection [65].

A majority of sexually active women contract HPV at least once in their lifetime. It is difficult to determine the absolute number of women who have ever had an HPV infection since not all infected individuals seroconvert or remain antibody positive. However, most infections are transient and get cleared spontaneously within a period of 2 years. There is evidence to indicate that the magnitude of antibody response after natural infection with HPV is significantly lower (~40-fold) than after vaccination [66,67,68,69]. Data from the Costa Rican HPV vaccine study—a randomized trial of the efficacy of the 2vHPV vaccine for prevention of HPV 16/18 infection and precancerous lesions—suggested that high levels of naturally induced antibodies can protect from re-infection. Among unvaccinated women, in the control arm of the vaccine trial, those with higher antibody titers to HPV16 and HPV18 were at a significantly reduced risk of subsequent HPV16 (50% reduction) and HPV18 (64% reduction) infection relative to seronegative women, for up to 4 years of follow-up [70]. However, it is still not conclusive if natural infection could evoke a response robust enough to trigger a cascade that could end up in generation and maintenance of a plethora of memory B cells that could result in long-term protection. Our lab has in the past studied the quality of B-cell response among the previously infected women after a single dose of HPV vaccine. The study demonstrated that while antibodies cloned from naturally elicited Bmem cells were generally non-neutralizing, those produced after a single dose of vaccination were both neutralizing and of higher titer [71]; thus, suggesting that sexually active persons also could potentially benefit from HPV vaccination. Women, previously exposed to HPV with detectable anti-HPV antibodies prior to vaccination, when immunized with quadrivalent vaccine, had ~12–26-fold higher titers of antibodies than HPV-naïve vaccine recipients [72], again suggesting the potential utility of vaccinating women who were HPV positive at baseline.

## 8. Lessons from Other Vaccines/Infection

While some vaccines produce lifelong immunity, others fail to do so. Smallpox vaccine, the first vaccine to be developed against an infectious disease that led to eradication of smallpox, has been shown to elicit decades-long immune responses in the form of vaccine-specific antibodies, memory B cells, and CD4(+) T cells [73,74]. Functional competence of Bmems specific to smallpox antigen has been established through demonstration of anamnestic response upon rechallenge with the antigen among the smallpox vaccine recipients several years later with a positive correlation observed between vaccine-specific circulating antibodies and Bmems [74]. Similarly, pertussis and measles vaccines, which are acellular and live-attenuated vaccines, respectively, mount an immune response with a positive correlation between circulating antibodies and Bmems [75]. The Hepatitis B surface antigen (HBsAg) is also arranged into VLPs, and when supplemented with adjuvant containing aluminum induces an immune response with detectable antibodies even after 10 years of immunization [76,77]. HBV antigen challenge of a substantial proportion of vaccine recipients lacking detectable levels of HBV vaccine-specific antibodies were shown to mount an anamnestic response by seroconverting rapidly for anti HBV antibodies, suggesting a strong Bmem response [78]. However, no correlation is observed between the frequency of HBsAg-specific Bmems and the corresponding serum antibody titer [79]. Observations/findings from studies that investigated immune responses to tetanus toxoid and diphtheria vaccine showed no significant linear correlation between the frequencies of circulating antigen-specific IgG-bearing memory B cells and the serum titers of antigen-specific IgG [80,81]. Tetanus-specific antibody titers remain stable after depletion of Bmems by removal of spleen and draining, indicating that Bmems are not required for maintenance of the antibody response itself [82]. Owing to the robust antigenic shift/drift in influenza viruses, there is a change in the viral strains every year/season. This along with the short-lived antibodies produced by vaccination makes it necessary that flu shots are taken each year. Influenza-specific bone marrow plasma cells (BMPCs) were detected 4 weeks after immunization with the seasonal inactivated influenza vaccine. However, the number of BMPCs returned to near their pre-vaccination levels after 1 year. Short life span of plasma cells induced by influenza vaccine accounts, in part, for the shorter protection span of the influenza vaccine [83].

There is compelling evidence for a stronger influence of LLPCs in establishing long-sustaining antibody response upon infection and/or vaccination rather than Bmems. The dissimilarity in the longevity of immunity seems to result from the difference in the ability of antigen included in the vaccine to produce long-lived plasmablasts. Here, we would like to reiterate the point that HPV vaccines induce stronger and longer antibody response than natural infection, which is quite unusual.

## 9. Summary and Open Questions

There are several factors that have contributed to the remarkable success of the HPV vaccine. The VLP is highly immunogenic and likely presents an array of epitopes that are closely packed for B cells to recognize. Additionally, most antibodies induced by VLPs are of high affinity and are neutralizing and, lastly, anti-HPV antibody titers persist many years after vaccination. While there are indications suggesting a single dose of a HPV vaccine provides good efficacy in preventing premalignant lesions, the titer of the vaccine-specific antibodies is lower than that elicited by two or three doses. Protection following one dose might be more dependent upon the Bmem response following exposure. However, there is no data available so far on Bmem response after a single dose of HPV vaccine. Neither have there been enough studies of local anamnestic responses following exposure. It would be a remarkable achievement in vaccinology if a single dose of an HPV vaccine is proven to generate a protective Bmem response upon re-exposure to the HPV antigens.

## Data Availability

Not applicable.
